# Impact of Alzheimer’s disease and related dementias on colorectal cancer screening utilization, knowledge, and associated health disparities

**DOI:** 10.3389/fphar.2022.872702

**Published:** 2022-09-07

**Authors:** Gang Lv, Xiaoxia Wang, Xiangxiang Jiang, Minghui Li, Kevin Lu

**Affiliations:** ^1^ Department of General Surgery, The First Medical Center of Chinese PLA General Hospital, Beijing, China; ^2^ College of Pharmacy, University of South Carolina, Columbia, SC, United States; ^3^ Department of Clinical Pharmacy and Outcomes Sciences, College of Pharmacy, University of South Carolina, Columbia, SC, United States; ^4^ Department of Clinical Pharmacy and Translational Science, University of Tennessee Health Science Center, Memphis, TN, United States

**Keywords:** Alzheimer’s disease, dementia, colorectal cancer screening, knowledge, health disparities

## Abstract

**Background:** Colorectal cancer screening can detect colorectal cancer at an early stage and reduce mortality. None of the existing clinical practice guidelines provide specific recommendations for colorectal cancer screening in patients with Alzheimer’s disease and related dementias (ADRD). Limited studies have assessed the impacts of ADRD on colorectal cancer screening use and knowledge, and no studies have focused on the associated health disparities.

**Objectives:** To examine the utilization, knowledge, and associated health disparities of colorectal cancer screening in older adults with ADRD.

**Methods:** This study used the Medicare Current Beneficiary Survey from 2015 to 2018. Two types of colorectal cancer screening, including fecal occult blood test (FOBT) and colonoscopy/sigmoidoscopy, were measured. The colorectal cancer screening knowledge was evaluated by asking if the participants have heard of two screening methods and whether they knew Medicare pays for colorectal cancer screenings. Logistic regression models were used to examine the impact of ADRD diagnosis on the utilization and knowledge of colorectal cancer screening.

**Results:** The overall colorectal cancer screening rate in older adults increased from 86.4% to 88.96% from 2015 to 2018. Patients with AD were 39% (OR: 0.61; 95% CI: 0.50–0.76) less likely and those with RD were 25% (OR: 0.75; 95% CI: 0.62–0.91) less likely to use any colorectal cancer screening when compared to older adults without ADRD. The rate of knowledge of colonoscopy/sigmoidoscopy remained high between 84.23% and 84.57% while the knowledge of FOBT increased from 64.32% to 78.69% during the study period. Compared to older adults without ADRD, those with AD were 77% (OR: 1.77; 95% CI: 1.12–2.81) more likely to hear of colonoscopy/sigmoidoscopy. The rate of knowledge of Medicare pay for colorectal cancer screening increased from 42.19% to 45.27% during the study period. Compared to older adults without ADRD, those with AD were 19% (OR: 0.81; 95% CI: 0.70–0.94) less likely to know that Medicare pays for colorectal cancer screening.

**Conclusion:** ADRD was significantly associated with colorectal cancer screening utilization and knowledge. In addition, this study identified health disparities in race/ethnicity, gender, and urban/rural residence in colorectal cancer screening use and knowledge.

## Introduction

In the United States, colorectal cancer is the third leading cause of cancer-related deaths, following lung cancer and breast cancer. Overall, the lifetime risk of developing colorectal cancer is about 4.3% for men and 4.0% for women. The American Cancer Society estimates that there will be 104,270 new cases of colon cancer and 45,230 new cases of rectal cancer in the United States in 2021, and the mortality rate in the same year is expected to be 52,980 cases (Siegel et al., 2021).

Unlike other types of cancers, colorectal cancers can be prevented through regular screening. A colorectal cancer screening can find precancerous polyps that can be removed before they turn into cancer. Additionally, a screening examination can detect colorectal cancer at an early stage when it is easier to treat and cure (US Preventive Services Task Force et al., 2016). To date, colonoscopy is the most sensitive and efficient screening method, which can detect and remove polys during the same procedure. According to the American College of Gastroenterology (ACG), cancer risk is reduced by 90 percent following a colonoscopy and the removal of precancerous polyps (Levin et al., 2018). In addition to colonoscopy, other screening exams are widely applied to screen colorectal cancers, including sigmoidoscopy and fecal occult blood test (FOBT). Unlike colonoscopy which examines the entire colon, sigmoidoscopy only scans the rectum and sigmoid colon. Some people choose sigmoidoscopy because it is less invasive and does not need a sedative, and the bowel prep is less complicated. Although sigmoidoscopy is less thorough than colonoscopy, these two screening methods are considered with high similarity because both are approved to reduce colorectal cancer rates and are covered by Medicare (Cardoso et al., 2019; Miller et al., 2019). FOBT is a noninvasive lab test used to check stool samples for hidden blood. Despite FOBT being the most convenient method when compared to other colorectal screening methods, it could show a false negative result because some cancers or polyps do not bleed (Li and Yuan, 2019).

It is well known that there is a close association between Alzheimer’s disease and related dementia (ADRD) and cancers. The risk of colorectal cancer increases as people get older. Most colorectal cancers occur in people older than 50, and the average age at the time of colorectal cancer diagnosis is 70 (Siegel et al., 2021)^,^ (Brenner et al., 2014). Cancer and ADRD are both diseases associated with aging, and it is not uncommon for older adults to have both conditions. A retrospective study found that a diagnosis of dementia prior to the diagnosis of colorectal cancer was associated with an increased risk of death by 45%; and the mean survival time for patients with advanced colorectal cancer and pre-existing dementia was 42% lower when compared with cognitively healthy counterparts (Chen et al., 2017).

To date, whether cancer screening is useful for patients with ADRD is a controversial topic. People living with ADRD usually suffer from comorbid illnesses, which result in poor functional status and reduced life expectancy (Libert et al., 2016). Many physicians and caregivers choose to forego chemotherapy for dementia patients with advanced colorectal cancer, especially in the later stages, because they do not want to further harm the patients with these aggressive treatments. However, increasing evidence showed that foregoing treatments were linked to worse survival in older patients with colorectal cancer who also had dementia (Yang et al., 2021). Some studies suggested that cancer screening should be avoided for patients with ADRD because of the shortened life expectancy, increased economic burden, and potential harms from the screening procedures (Torke et al., 2013; Van der Willik et al., 2018); while other evidence highly recommends colorectal cancer screening for patients with dementia because it may decrease the trend in colorectal cancer prevalence and mortality rate among this vulnerable population (Kotwal and Schonberg, 2017; Kuwata et al., 2021). Furthermore, several evidence-based guidelines, such as ACG and NCCN (National Comprehensive Cancer Network), support screening for a variety of cancer types, but none of the existing guidelines provide specific recommendations for patients with ADRD (Shaukat et al., 2021).

Lacking colorectal cancer screening knowledge is an important reason for people not using colorectal cancer screening. A study showed that 67.1% of eligible adults have received colorectal cancer screening in 2019, and the screening rate has continuously increased in recent decades. However, about one in three adults in the United States is not getting colorectal cancer screening as recommended by clinical guidelines, and current efforts are directed towards increasing the screening rate to 80% (Shaukat and Church, 2020). One of the important reasons for the low colorectal cancer screening rate could be the deficient knowledge about the importance of colorectal cancer screening as well as insurance coverage (Brajcich et al., 2022; Carnahan et al., 2021). It is possible that patients with ADRD might not receive adequate information about colorectal cancer screening because their physicians might not recommend this exam due to their underlying comorbidities and cognitive function. Moreover, some ADRD patients and caregivers might not know that most insurances cover the cost of colorectal cancer screening, and they might avoid the screening examination due to the worry of financial burden.

Barriers to colorectal cancer screening are multifaceted, social determinants of health, such as age, gender, race/ethnicity, residence, socioeconomic status, and income level, may influence colorectal screening use and knowledge (Brajcich et al., 2022). One study showed that non-Hispanic black males had lower rates of colorectal cancer screening use and less knowledge when compared to non-Hispanic white males (Carnahan et al., 2021). Another study indicated that gender disparities in colorectal cancer screening use persisted even among the insured, educated, and high-income population group, and females were less likely to undergo colorectal cancer screening when compared to males (Yager and Cheung, 2011). Moreover, people with lower levels of education were found to have greater difficulties making an informed clinical decision and lower participant rates in colorectal cancer screening (Smith et al., 2014). Overall, these social determinants of health may result in low survival rates and poor clinical outcomes in those not having colorectal cancer screening.

Although a growing number of studies demonstrated the gender and racial/ethnic disparities in colorectal cancer screening use, there are limited studies focused on investigating the impacts of ADRD on colorectal cancer screening use and knowledge. No studies have focused on investigating the health disparities of colorectal cancer screening knowledge and use in patients with ADRD. Herein, there is an urgent need to identify the predictors of colorectal cancer screening use and knowledge among the ADRD population. The goal of this study is to examine the trends in colorectal cancer screening use and knowledge among older adults and investigate the impact of ADRD on colorectal cancer screening use and knowledge. Moreover, we study the potential health disparities in colorectal cancer screening use and knowledge.

## Methods

### Data source

The Medicare claims and Medicare Current Beneficiary Survey (MCBS) linked data from 2015 to 2018 were used for this cross-sectional study. Medicare is federal health insurance covering older adults and younger adults with certain disabilities in the United States. Medicare claims include Part A inpatient, Part B outpatient, and Part D prescription information. The MCBS is a nationally representative survey of Medicare beneficiaries conducted by the Centers for Medicare and Medicaid Services (CMS). The MCBS data include information on demographics, socioeconomics, health status, knowledge, health insurance, medical conditions, health care utilization, and expenditures. The linked data provide comprehensive information on preventive care utilization and knowledge related to colorectal cancer. Medicare beneficiaries who were 65 years and older and responded to the MCBS were included in this study. Those who have been diagnosed with colorectal cancer were excluded from this study. To ensure the reliability of the data for some patients (e.g., ADRD), if the interviewer and the caregiver determine that the interviewee is not able to provide reliable data, a “proxy”, generally the caregiver of the patient, or a family member, such as a spouse, will answer the questions on behalf of the patient to ensure the quality of the data. The quality of the MCBS data have been validated in the past 30 years with the over 1 million interviews.

### Measurement

The study population was classified as Medicare beneficiaries without ADRD, patients with Alzheimer’s disease (AD), and patients with related dementias (RD) based on self-reported information on chronic conditions. The colorectal cancer screening was measured based on preventive health behaviors. Two types of colorectal cancer screening, FOBT and colonoscopy/sigmoidoscopy, were measured in this study. If study participants had either home FOBT or colonoscopy/sigmoidoscopy, they will be classified as having colorectal cancer screening. This study also measured knowledge on colorectal cancer screening. Study participants were asked if they have heard of home FOBT or colonoscopy/sigmoidoscopy. They were further asked if they knew Medicare pays for colorectal cancer screening. Demographic and socioeconomic characteristics were measured based on self-reports. Due to the progression of AD and RD and delined mental status of some patients, some participants were facilitated by their caregivers to answer the self-report surveys.

### Statistical analysis

The Chi-square tests were used to compare the differences in demographic and socioeconomic characteristics, the utilization of colorectal cancer screening, and the knowledge of colorectal cancer screening among Medicare beneficiaries without ADRD, patients with AD, and patients with RD. The change in the use and knowledge of colorectal cancer screening was plotted over time. We further plotted the composition of the use and knowledge of colorectal cancer screening by certain characteristics, including gender, race/ethnicity, and urban/rural residence. Adjusted logistic regression models were used to examine the impact of ADRD diagnosis on the utilization and knowledge of colorectal cancer screening by controlling for potential confounding variables. To generate national estimates, survey sampling weights were used in this study.

## Results

After applying the survey sampling weights, 178, 820, 457 older adults without colorectal cancer were included in this study, among which 3,394,759 (1.90%) had AD and 4,067,948 (2.27%) had RD. Compared to older adults without ADRD, those with AD and RD were significantly different in age, race/ethnicity, education level, marital status, and income (all *p* < 0.05). No significant differences were found in gender (*p* = 0.0568), residence status (*p* = 0.2143), and census region (*p* = 0.5197) between older adults with and without ADRD (Table 1).

**TABLE 1:** Characteristics of older adults without colorectal cancer.

	Overall	ADRD			
		**No**	**AD**	**RD**	P
	*n* = 178,820,457	*n* = 166,116,241	*n* = 3,394,759	*n* = 4,067,948	
	%	%	%	%	
Age					<.0001
65–74	55.94	58.38	26.19	27.19	
75–84	31.09	30.83	39.48	37.72	
85+	12.97	10.79	34.34	35.09	
Gender					0.0568
Female	56.02	55.40	60.09	58.81	
Male	43.98	44.60	39.91	41.19	
Race/ethnicity					<.0001
Non-Hispanic white	77.38	77.56	65.46	71.52	
Non-Hispanic black	8.49	8.39	13.43	9.42	
Hispanic	8.04	7.95	11.98	11.80	
Other	6.09	6.09	9.13	7.26	
Education					<.0001
Less than high school	15.47	14.66	30.86	27.33	
High school graduate	25.69	25.34	30.32	26.96	
Some college	23.50	23.90	15.89	19.40	
College graduate	35.34	36.10	22.93	26.31	
Marital status					<.0001
Married	55.32	56.84	48.65	46.70	
Widowed	24.35	22.88	35.71	36.18	
Single	20.33	20.28	15.64	17.12	
Income					<.0001
Less than $10,000	7.04	6.35	14.53	12.34	
$10,000 - $19,999	17.88	16.92	26.31	23.83	
$20,000 - $39,999	26.81	26.91	25.16	29.61	
$40,000 and more	48.27	49.82	34.00	34.22	
Residence					0.2143
Urban	80.35	80.36	78.55	83.12	
Rural	19.65	19.64	21.45	16.88	
Census region					0.5197
Northeast	18.53	18.50	15.37	19.64	
Midwest	22.28	22.11	24.38	23.83	
South	37.42	37.54	39.23	34.55	
West	21.77	21.85	21.02	21.98	
Screening use					
FOBT					<.0001
No	47.37	46.91	58.19	58.33	
Yes	52.63	53.09	41.81	41.67	
Colonoscopy/sigmiodoscopy					<.0001
No	21.49	21.09	32.76	29.37	
Yes	78.51	78.91	67.24	70.63	
Any colorectal cancer screening					<.0001
No	12.14	11.74	23.54	19.68	
Yes	87.86	88.26	76.46	80.32	
Screening knowledge					
Heard of FOBT					<.0001
No	27.26	26.88	31.20	35.62	
Yes	72.74	73.12	68.80	64.38	
Heard of colonoscopy/sigmoidoscopy					0.2400
No	15.36	15.27	12.82	19.02	
Yes	84.64	84.73	87.18	80.98	
Medicare pay for colorectal cancer screening					<.0001
No	55.20	54.90	62.78	61.35	
Yes	44.80	45.10	37.22	38.65	

**FIGURE 1:**
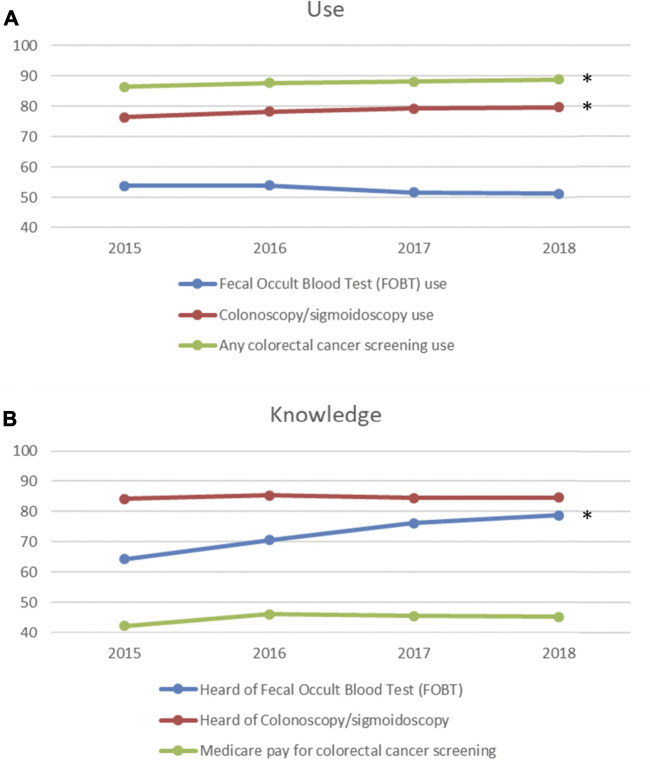
Trends in colorectal cancer screening use and knowledge **(A)**. Trends in colorectal cancer screening use; **(B)**. Trends in colorectal cancer screening knowledge). *: significant at .05 level.

The overall colorectal cancer screening rate was high and significantly increased from 86.40% to 88.96% (*p* = 0.0328) from 2015 to 2018 (Figure 1A). For different types of colorectal cancer screening, the screening rate of colonoscopy/sigmoidoscopy was high and significantly increased from 76.44% to 79.78% (*p* = 0.0411), but the screening rate of FOBT was medium and remained stable from 53.83% to 51.17% (*p* = 0.0869) during the study period (Figure 1A). AD and RD were significantly associated with any colorectal cancer screening use. Compared to older adults without ADRD, those with AD were 39% (OR: 0.61; 95% CI: 0.50–0.76) less likely and those with RD were 25% (OR: 0.75; 95% CI: 0.62–0.91) less likely to use any colorectal cancer screening (Table 2). Only AD was significantly associated with colonoscopy/sigmoidoscopy use. Compared to older adults without ADRD, those with AD were 23% (OR: 0.77; 95% CI: 0.64–0.94) less likely to use colonoscopy/sigmoidoscopy (Table 2). For FOBT use, AD patients were 30% (OR: 0.70; 95% CI: 0.58–0.84) less likely and RD patients were 31% (OR: 0.69; 95% CI: 0.61–0.78) less likely to use FOBT (Table 2).

**TABLE 2:** Factors associated with colorectal cancer screening use.

	Fecal Occult Blood Test (FOBT) use			Colonoscopy/sigmoidoscopy use			Any colorectal cancer screening use		
	OR	95% CI		OR	95% CI		OR	95% CI	
ADRD									
No	Ref								
AD	0.70	0.58	0.84	0.77	0.64	0.94	0.61	0.50	0.76
RD	0.69	0.61	0.78	0.85	0.68	1.06	0.75	0.62	0.91
Age									
65–74	Ref								
75–84	1.13	1.07	1.21	1.07	0.98	1.17	1.00	0.91	1.10
85+	0.94	0.87	1.01	0.76	0.67	0.86	0.68	0.60	0.76
Gender									
Female	Ref								
Male	0.91	0.86	0.97	0.94	0.87	1.02	0.87	0.79	0.95
Race/ethnicity									
Non-Hispanic white	Ref								
Non-Hispanic black	0.95	0.83	1.08	1.07	0.92	1.25	1.01	0.84	1.22
Hispanic	1.07	0.86	1.33	0.94	0.80	1.11	1.06	0.85	1.33
Other	0.79	0.69	0.90	0.61	0.50	0.73	0.56	0.46	0.69
Education									
Less than high school	Ref								
High school graduate	1.11	1.01	1.22	1.38	1.22	1.57	1.39	1.21	1.59
Some college	1.25	1.13	1.38	1.76	1.53	2.03	1.65	1.41	1.94
College graduate	1.33	1.19	1.48	2.11	1.86	2.40	2.01	1.70	2.38
Marital status									
Married	Ref								
Widowed	0.94	0.87	1.01	0.86	0.78	0.96	0.80	0.72	0.90
Single	0.99	0.90	1.09	0.76	0.67	0.87	0.81	0.70	0.94
Income									
Less than $10,000	Ref								
$10,000 - $19,999	1.07	0.96	1.20	1.26	1.11	1.43	1.25	1.09	1.45
$20,000 - $39,999	1.28	1.14	1.44	1.74	1.54	1.97	1.76	1.55	2.01
$40,000 and more	1.34	1.16	1.55	2.58	2.23	2.98	2.71	2.30	3.19
Residence									
Urban	Ref								
Rural	0.84	0.69	1.03	0.83	0.75	0.93	0.72	0.60	0.86
Census region									
Northeast	Ref								
Midwest	1.15	0.96	1.38	1.03	0.86	1.23	1.14	0.92	1.41
South	1.29	1.11	1.50	0.97	0.84	1.13	1.09	0.89	1.33
West	1.75	1.37	2.24	0.78	0.65	0.94	1.02	0.78	1.35

FOBT, Fecal Occult Blood Test; ADRD, Alzheimer’s disease and related dementias; CI, confidence interval

The rate of the knowledge of Medicare pay for colorectal cancer screening was medium and remained stable from 42.19% to 45.27% (*p* = 0.3590) from 2015 to 2018 (Figure 1B). The rate of knowledge of FOBT significantly increased from 64.32% to 78.69% (*p* = 0.0160), while the rate of knowledge of colonoscopy/sigmoidoscopy was high and remained stable between 84.23% and 84.57% (*p* = 0.9534) during the study period (Figure 1B). AD and RD were found to be not associated with the knowledge of FOBT. Factors associated with the knowledge of FOBT included age, gender, race/ethnicity, education level, income, and residence status (Table 3). After adjusting for variables, we found that AD was significantly associated with the knowledge of colonoscopy/sigmoidoscopy. Compared to older adults without ADRD, those with AD were 77% (OR: 1.77; 95% CI: 1.12–2.81) more likely to hear of colonoscopy/sigmoidoscopy. In addition, AD was significantly associated with the knowledge of Medicare pay for colorectal cancer screening. Compared to older adults without ADRD, those with AD were 19% (OR: 0.81; 95% CI: 0.70–0.94) less likely to know that Medicare pays for colorectal cancer screening (Table 3).

**TABLE 3:** Factors associated with colorectal cancer screening knowledge.

	Heard of Fecal Occult Blood Test (FOBT)			Heard of Colonoscopy/sigmoidoscopy			Medicare pay for colorectal cancer screening		
	OR	95% CI		OR	95% CI		OR	95% CI	
ADRD									
No	Ref								
AD	1.14	0.89	1.47	1.77	1.12	2.81	0.81	0.70	0.94
RD	0.87	0.69	1.09	1.03	0.70	1.53	0.86	0.74	1.01
Age									
65–74	Ref								
75–84	0.77	0.71	0.84	0.69	0.57	0.82	1.06	1.00	1.12
85+	0.57	0.50	0.64	0.60	0.48	0.75	0.80	0.73	0.88
Gender									
Female	Ref								
Male	0.62	0.56	0.69	0.61	0.52	0.72	0.85	0.80	0.90
Race/ethnicity									
Non-Hispanic white	Ref								
Non-Hispanic black	0.81	0.67	0.99	0.48	0.37	0.62	0.75	0.65	0.85
Hispanic	0.46	0.37	0.57	0.52	0.38	0.71	0.94	0.83	1.07
Other	0.46	0.37	0.58	0.35	0.26	0.47	0.77	0.68	0.87
Education									
Less than high school	Ref								
High school graduate	1.42	1.27	1.60	1.49	1.19	1.85	1.06	0.98	1.15
Some college	1.59	1.38	1.84	1.95	1.44	2.64	1.21	1.09	1.34
College graduate	1.65	1.42	1.92	2.17	1.51	3.12	1.39	1.25	1.54
Marital status									
Married	Ref								
Widowed	0.90	0.79	1.01	0.86	0.70	1.06	0.85	0.79	0.90
Single	0.90	0.80	1.00	0.85	0.66	1.11	0.88	0.81	0.96
Income									
Less than $10,000	Ref								
$10,000 - $19,999	1.06	0.90	1.25	1.01	0.79	1.30	1.02	0.89	1.16
$20,000 - $39,999	1.36	1.15	1.61	1.43	1.07	1.92	1.02	0.89	1.17
$40,000 and more	1.62	1.36	1.93	1.91	1.41	2.60	1.04	0.91	1.20
Residence									
Urban	Ref								
Rural	1.22	1.07	1.39	1.17	0.95	1.42	1.00	0.90	1.12
Census region									
Northeast	Ref								
Midwest	0.89	0.71	1.13	0.73	0.56	0.94	0.85	0.74	0.98
South	1.03	0.91	1.18	0.77	0.61	0.98	0.95	0.81	1.11
West	0.88	0.75	1.02	0.71	0.56	0.91	0.80	0.68	0.94

FOBT, Fecal Occult Blood Test; ADRD, Alzheimer’s disease and related dementias; CI, confidence interval

**FIGURE 2:**
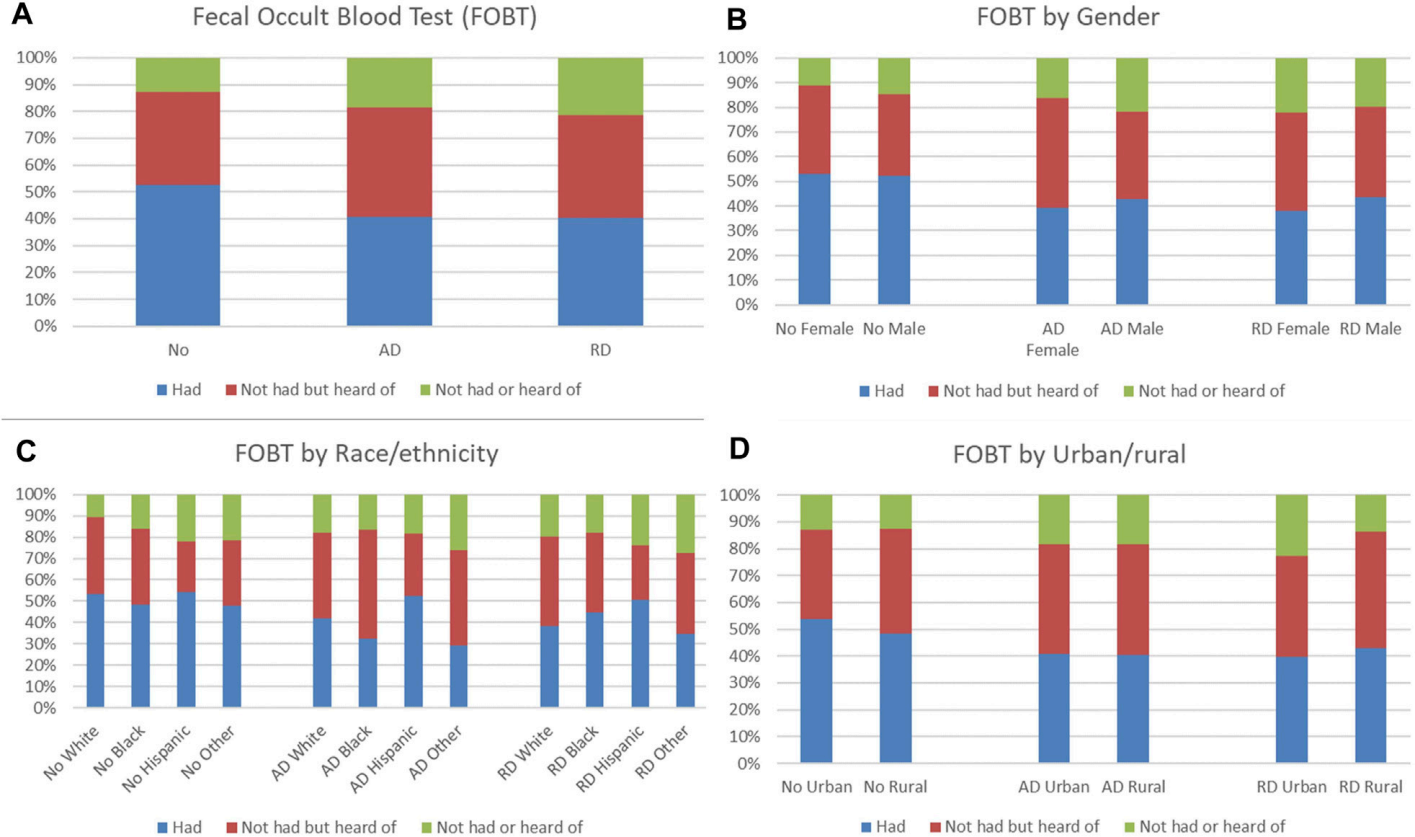
Distribution of fecal occult blood test (FOBT) use and knowledge by ADRD status **(A)**. Overall distribution; **(B)**. Distribution by gender; **(C)**. Distribution by race/ethnicity; **(D)**. Distribution by urban/rural).

**FIGURE 3:**
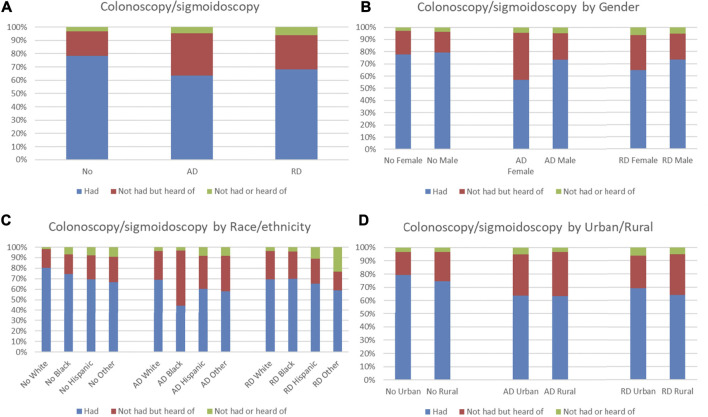
Distribution of colonoscopy/sigmoidoscopy use and knowledge by ADRD status **(A)**. Overall distribution; **(B)**. Distribution by gender; **(C)**. Distribution by race/ethnicity; **(D)**. Distribution by urban/rural).

Distributions of FOBT and colonoscopy/sigmoidoscopy use and knowledge were compared by ADRD status. Among older adults without ADRD, 52.71% had FOBT, which was higher than those with AD (40.74%) and with RD (40.22%), but 12.71% had not had or heard of FOBT, which was lower than those with AD (18.49%) and with RD (21.29%) (Figure 2A). Males had a higher proportion of having FOBT than females in those with ADRD (Figure 2B). For race/ethnicity, Hispanics had the highest proportion of having FOBT in those with ADRD (Figure 2C). No obvious patterns in distributions of FOBT use and knowledge were observed in those with ADRD in terms of urban/rural residence (Figure 2D). Among older adults without ADRD, 78.33% had colonoscopy/sigmoidoscopy, which was higher than those with AD (63.57%) and with RD (68.31%), but 3.31% had not had or heard of colonoscopy/sigmoidoscopy, which was lower than those with AD (4.67%) and with RD (6.03%) (Figure 3A). Males had a higher proportion of having colonoscopy/sigmoidoscopy than females in those with ADRD (Figure 3B). No obvious patterns in distributions of colonoscopy/sigmoidoscopy use and knowledge were observed in those with ADRD in terms of race/ethnicity (Figure 3C). Urban residents had a higher proportion of not having or hearing colonoscopy/sigmoidoscopy than rural residents in those with ADRD (Figure 3D).

**FIGURE 4:**
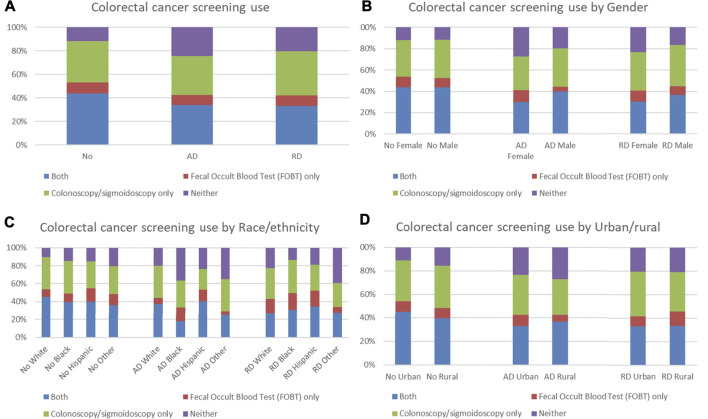
Distribution of types of colorectal cancer screening use by ADRD status **(A)**. Overall distribution; **(B)**. Distribution by gender; **(C)**. Distribution by race/ethnicity; **(D)**. Distribution by urban/rural).

We further compared distributions of types of colorectal cancer screening by ADRD status. Among older adults without ADRD, 11.82% did not have any colorectal cancer screening, which was lower than those with AD (24.16%) and with RD (20.40%) (Figure 4A). Females had a higher proportion of not having any colorectal cancer screening than males in those with ADRD (Figure 4B). No obvious patterns in distributions of types of colorectal cancer screening were observed in those with ADRD in terms of race/ethnicity and urban/rural residence (Figures 4C,D).

## Discussion

This study found an increasing trend in the knowledge of FOBT but a decreasing trend in the use of FOBT. Although FOBT is noninvasive and convenient, the decreasing trend in the use of FOBT may be explained by the lower sensitivity and efficiency compared to direct visual exams. Moreover, older adults might be aware of the limitations of FOBT that a further colonoscopy/sigmoidoscopy is needed if FOBT shows a positive result (Li and Yuan, 2019). This study also found a stable trend in the knowledge of colonoscopy/sigmoidoscopy and an increasing trend in the use of colonoscopy/sigmoidoscopy. The high rate of knowledge of colonoscopy/sigmoidoscopy supports the elevation in the utilization of colonoscopy/sigmoidoscopy. Noticeably, our results showed that less than half of older adults knew that Medicare pays for colorectal cancer screening. Different stakeholders, including healthcare providers, payers, and policymakers, should make an effort to help people understand the medical coverage and payment of colorectal cancer screening. Although most people receive information from their providers and had a good awareness of colorectal cancer screening, some people might choose to avoid colorectal cancer screening due to the fear of financial burden.

ADRD is an important factor associated with colorectal cancer screening use and knowledge. Older adults with ADRD are less likely to use colorectal cancer screening compared to those without ADRD. Our results are consistent with recent publications that many physicians do not recommend colorectal cancer screening for patients with dementia due to their reduced life expectancy, and patients with dementia may be unnecessarily harmed by the burden of screening procedures (Torke et al., 2013; Van der Willik et al., 2018). However, it is still controversial whether patients with ADRD should get colorectal cancer screening, and there are no clear recommendations from clinical guidelines (Davidson et al., 2021; Shaukat et al., 2021). Moreover, older adults with AD are more likely to know about colonoscopy/sigmoidoscopy but less likely to know about Medicare coverage of colorectal cancer screening. Our finding confirmed that older adults suffering from ADRD have a lower rate of colorectal cancer screening even though most of them have adequate knowledge of the importance of colorectal cancer screening. The underlying reason might be attributed to low awareness of Medicare coverage. Considering an expected increase in the prevalence of ADRD over the next few decades, our study demonstrated that it is increasingly important to ensure that clinical practice guidelines adequately reflect the needs of ADRD patients and raise awareness of insurance coverage for both ADRD patients and their caregivers.

This study found that Medicare beneficiaries older than 85 years are less likely to have colorectal cancer screening. This result is consistent with the current ACG and USPSTF clinical guidelines that recommend against routine cancer screening for colorectal cancer in adults aged 76 and older (Davidson et al., 2021). However, clinical studies showed that people who had colorectal cancer screening after age 75 had a lower risk of colorectal cancer and a lower risk of death from colorectal cancer (Ma et al., 2021). Due to this reason, individuals older than 75 years of age may be more likely to get FOBT, which is a noninvasive test and can be performed at home.

Health disparities in race/ethnicity, gender, and urban/rural residence have been identified in colorectal cancer screening use and knowledge. Racial/ethnic minorities are less likely to have colorectal cancer screening and related knowledge. The low rate of colorectal cancer screening in racial/ethnic minorities might be attributed to deficient knowledge. The observed racial/ethnic disparities might be contributed to the language barriers, preventive care use, and access barriers (Meester et al., 2018). Healthcare professionals should be aware of the low utilization and knowledge of colorectal cancer screening in racial/ethnic minorities. Greater efforts are needed to reduce racial/ethnic disparities, including providing cancer screening knowledge in various types of languages, offering translation services and more education for people with a lower level of health literacy, and providing transportation. This study identified gender disparities in the use and knowledge of colorectal cancer screening. Our analysis showed that males had a higher proportion of having FOBT than females in ADRD patients. This finding is consistent with previous evidence that women are less likely to be screened for colorectal cancer than men (Friedemann-Sánchez et al., 2007). Although the underlying reasons for these gender disparities are unknown, it is possible that screening in females is less efficient because women have more right-sided cancers, which are more difficult to find (Hultcrantz, 2021). Moreover, females have a lower incidence of colorectal cancer than males, which might be another reason for the lower screening rates (Chacko et al., 2015). These gender disparities recommend physicians focus more on providing colorectal cancer screening education to female patients. In addition, this study found urban/rural disparities in colonoscopy/sigmoidoscopy but not in FOBT use. This might be due to the transportation and healthcare access barriers to colonoscopy/sigmoidoscopy in rural areas, and more rural residents prefer FOBT because it could be performed in their houses rather than clinics. Additionally, another underlying reason might be the varied healthcare quality between urban and rural areas (Anderson et al., 2013; Shete et al., 2021). As physicians are scarce in rural and underserved areas, individualized interventions to improve colorectal cancer screening quality are needed in rural areas.

To our knowledge, this is the first study to investigate colorectal cancer screening use and knowledge in ADRD. This study has some limitations. First, this is a cross-sectional study. Thus, this study cannot determine the causal relationship between ADRD and colorectal cancer screening use and knowledge. Second, colorectal cancer screening use and knowledge are based on survey responses and are subject to reporting bias. Third, this study is subject to proxy response bias. Some caregivers respond to the survey on behalf of participants because of the limited cognitive function of some ADRD patients. Although some participants’ self-report surveys were completed under caregivers’ guidance, this method increased the response rates and relative accuracy, which helps us better understand the association among cancer screening utilization, knowledge, and ADRD.

## Conclusion

ADRD was significantly associated with colorectal cancer screening utilization and knowledge. In addition, this study identified health disparities in race/ethnicity, gender, and urban/rural residence in colorectal cancer screening use and knowledge. Our study provides real-world evidence to suggest that clinical professionals should educate ADRD patients and their caregivers on the benefits and risks associated with colorectal cancer screening. Special attention should be paid to the minority populations of non-Hispanic blacks, Hispanics, females, and rural residents. There is also an urgent need for clear clinical guidance regarding colorectal cancer screening for older adults with ADRD.

## Data Availability

The data analyzed in this study is subject to the following licenses/restrictions: The data will not be made readily available for the privacy of the participants. Requests to access these datasets should be directed to and https://www.cms.gov/Research-Statistics-Data-and-Systems/Research/MCBS.
